# Assessment of Polyethylene Glycol-Coated Gold Nanoparticle Toxicity and Inflammation In Vivo Using NF-κB Reporter Mice

**DOI:** 10.3390/ijms21218158

**Published:** 2020-10-31

**Authors:** Tzu-Yin Chen, Mei-Ru Chen, Shan-Wen Liu, Jin-Yan Lin, Ya-Ting Yang, Hsin-Ying Huang, Jen-Kun Chen, Chung-Shi Yang, Kurt Ming-Chao Lin

**Affiliations:** 1 Institute of Biomedical Engineering and Nanomedicine, National Health Research Institutes, Zhunan, Miaoli 35053, Taiwan; chenty@nhri.edu.tw (T.-Y.C.); 940501@nhri.edu.tw (M.-R.C.); jwliu@nhri.edu.tw (S.-W.L.); k552999@nhri.edu.tw (J.-Y.L.); amyting@nhri.edu.tw (Y.-T.Y.); sing@nhri.edu.tw (H.-Y.H.); jkchen@nhri.edu.tw (J.-K.C.); cyang@nhri.edu.tw (C.-S.Y.); 2 Institute of Population Health, National Health Research Institutes, Zhunan, Miaoli 35053, Taiwan

**Keywords:** gold nanoparticle, PEG surface modification, liver inflammation, reporter imaging, NF-κB, steatosis

## Abstract

Polyethylene glycol (PEG) coating of gold nanoparticles (AuNPs) improves AuNP distribution via blood circulation. The use of PEG-coated AuNPs was shown to result in acute injuries to the liver, kidney, and spleen, but long-term toxicity has not been well studied. In this study, we investigated reporter induction for up to 90 days in NF-κB transgenic reporter mice following intravenous injection of PEG-coated AuNPs. The results of different doses (1 and 4 μg AuNPs per gram of body weight), particle sizes (13 nm and 30 nm), and PEG surfaces (methoxyl- or carboxymethyl-PEG 5 kDa) were compared. The data showed up to 7-fold NF-κB reporter induction in mouse liver from 3 h to 7 d post PEG-AuNP injection compared to saline-injected control mice, and gradual reduction to a level similar to control by 90 days. Agglomerates of PEG-AuNPs were detected in liver Kupffer cells, but neither gross pathological abnormality in liver sections nor increased activity of liver enzymes were found at 90 days. Injection of PEG-AuNPs led to an increase in collagen in liver sections and elevated total serum cholesterol, although still within the normal range, suggesting that inflammation resulted in mild fibrosis and affected hepatic function. Administrating PEG-AuNPs inevitably results in nanoparticles entrapped in the liver; thus, further investigation is required to fully assess the long-term impacts by PEG-AuNPs on liver health.

## 1. Introduction

Investigation and development of the therapeutic use of metal-based nanoparticles in drug delivery and imaging have been pursued for decades, but various challenges remain. The most common strategies to enhance distribution, prevent aggregation, and reduce the toxicity of nanoparticles include coating the particles with polymers such as polyethylene glycol (PEG), and changing the size and shape of the nanoparticles [1,2,3,4,5]. Most nanoparticles, including gold nanoparticles (AuNPs), are rapidly sequestered by the mononuclear phagocyte system (MPS), which includes blood monocytic and Kupffer cells in the liver, macrophages in the spleen and lymph nodes, and liver sinusoidal endothelial cells of the reticuloendothelial system (RES). High clearing efficiency by the MPS and RES contributes to low nanoparticle delivery to a target site (e.g., tumor), while PEG coating can increase the half-life in circulation and dose of AuNPs at the target site by allowing nanoparticles to avoid clearing by MPS and RES [4,6]. The degree of PEGylation alters the basic physiochemical properties of AuNPs and impacts uptakes by immune cells [7]. Adsorption of serum proteins by PEGylated AuNPs and the efficiency of uptake by macrophages have been shown to be particle size and PEG density-dependent [4,8].

Toxicity of AuNPs has been studied in the context of acute inflammation, compromised immune response, and liver and kidney toxicity [9]. The majority of AuNPs accumulate in hepatic and splenic tissues, and this is observed even at 6 months (mos) post-administration [10]. Most short-term studies have shown that a PEG coating significantly reduces the toxicity of AuNPs, as has been demonstrated from several days up to 1 month (mo) post-administration [1,11,12]. In liver and other tissues that host the MPS, whether accumulation of PEGylated AuNPs results in sustained inflammation beyond 1 mo, causing delayed or chronic toxicity, has not been fully characterized [7,9,13]. Moreover, whether chronic toxicity due to AuNPs can be detected by a standard pathology examination or requires a more sensitive method was not addressed before this study.

Molecular imaging techniques and reporter genes have enabled longitudinal monitoring of molecular events in live animals that are otherwise difficult to study. This technology allows tracing and quantification of biological processes in a long-term study without the need for sacrificing animals at each time point, thereby reducing the use of experimental animals. Signaling cascades mediating the cell responses to inflammation, wound healing, stress response, cell death, and regeneration often converge to activate transcription factor NF-κB, which stimulates the transcription of many proinflammatory genes and inflammatory intermediators. The bioluminescent NF-κB reporter transgenic (Tg) mouse is one of the earliest generated and most widely utilized imaging reporter mouse models [14,15,16,17,18], in which activation of NF-κB induces expression of the firefly luciferase gene and subsequent protein production in affected cells [14,15,16,17,18]. Detection of bioluminescence is used as a surrogate marker for in situ NF-κB pathway activation.

Whether the accumulation of PEG-coated AuNPs in hepatic tissues causes long-term toxicity in the liver has not been investigated. In this study, we coated AuNPs with methoxyl capped PEG (mPEG) or carboxymethyl capped PEG (cPEG), intravenously injected the nanoparticles into NF-κB reporter Tg mice, and monitored reporter protein production for 3 mos following injection. We showed that the PEG-AuNPs increased the bioluminescence in liver tissue up to 7-fold above that of saline-injected control mice from 3 h to 7 days post-administration, indicating that PEG-AuNPs induced NF-κB activation in liver, and that the induction was dependent on the size and surface modification of the AuNPs.

## 2. Results

### 2.1. NF-κB Reporter Mouse and In Vivo Response to Inflammation-Inducing Agents

**Figure 1:**
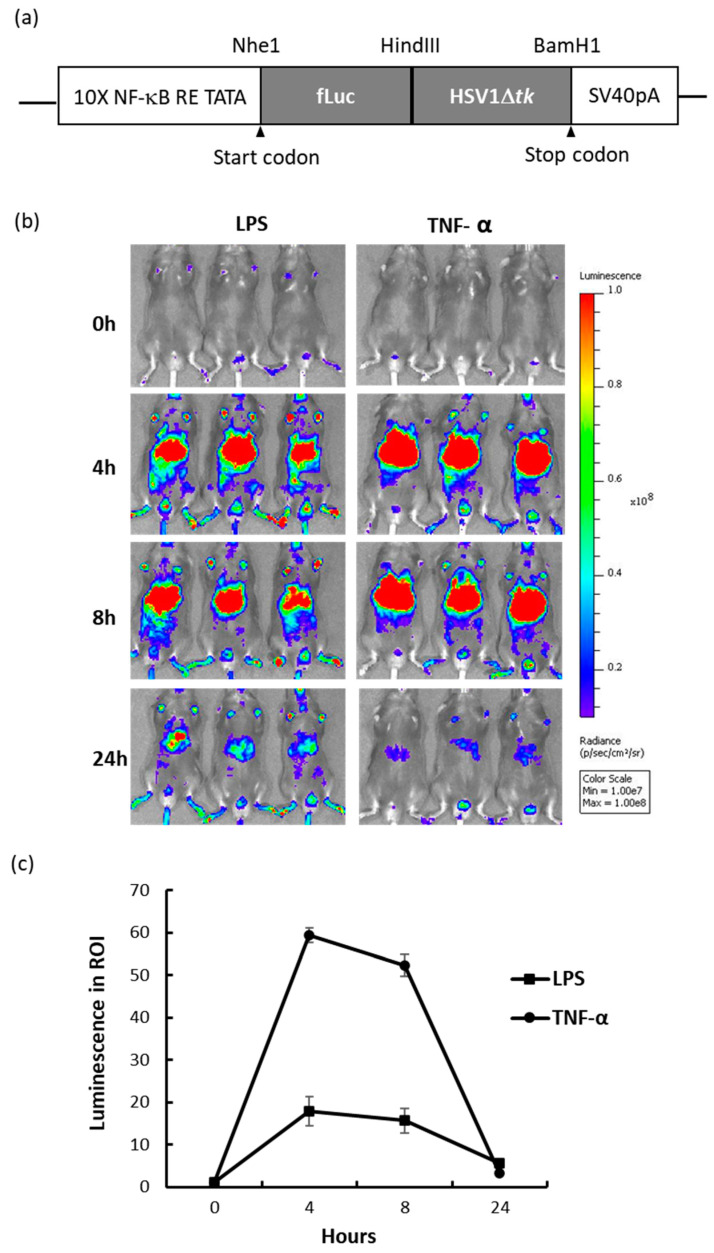
NF-κB dual reporter mouse and in vivo response to LPS and TNF-α treatment. (**a**) Schematic of the transgenic vector (not to scale). An NF-κB responsive promoter was placed before the coding sequence of firefly luciferase (fLuc) and the herpes simplex virus truncated thymidine kinase (HSV1Δtk) fusion protein. (**b**) Bioluminescence imaging of reporter mice before (0 h), and at 4 h, 8 h, and 24 h after i.p. injection of LPS (1 mg/Kg) or TNF-α (2 μg). (**c**) Fold induction of bioluminescence by LPS or TNF-α within the abdominal region of interest (ROI) compared to pre-injection values; *n* = 3. LPS, lipopolysaccharide.

NF-κB reporter mice were constructed by designing a fusion reporter vector that included firefly luciferase (fLuc) and herpes simplex virus truncated thymidine kinase (HSV1Δtk) under the control of NF-κB responsive elements. The schematic of the Tg vector is shown in Figure 1a; ten copies of NF-κB responsive elements were used to drive reporter expression. The Tg reporter mouse was designed to allow flexibility in use of either optical imaging (bioluminescence) or nuclear imaging (PET, SPECT) platforms, and the detectability of HSV1Δtk via nuclear imaging had been previously validated [19]. To assess the response of the NF-κB reporter to inflammation-inducing agents, Tg mice were injected intraperitoneally (i.p.) with endotoxin (LPS, 1 mg/kg body weight) or TNF-α (2 μg), and the resulting bioluminescence was measured for 24 h. Treatment with LPS and TNF-α resulted in 20 and 60-fold bioluminescence induction after 4 h, respectively, in the livers of Tg mice (Figure 1b,c). After 4 h, the bioluminescence decreased and remained at least 3 to 5-fold higher than the background level at 24 h. These results provided validation that the NF-κB reporter mouse was responsive to inflammatory stimuli, such as LPS and TNF-α, via in situ NF-κB reporter gene activation [18].

### 2.2. mPEG- and cPEG-AuNPs

**Figure 2:**
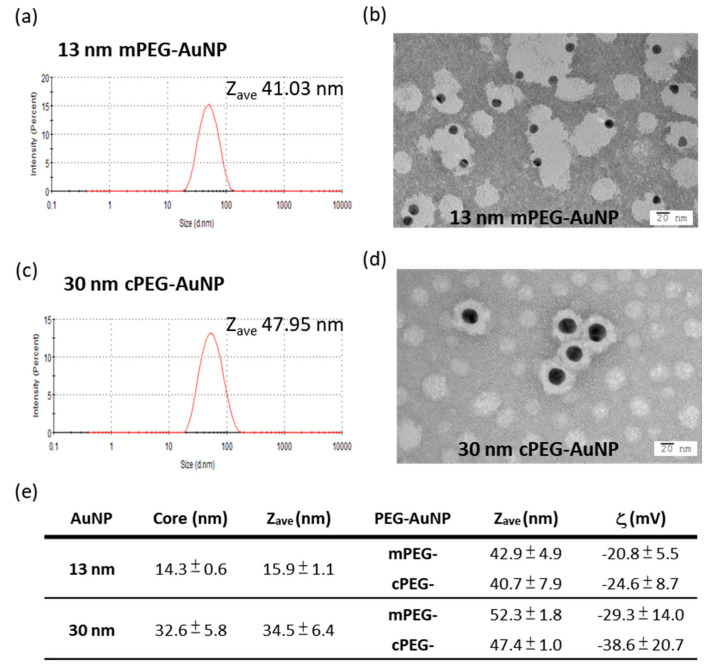
Hydrodynamic and core sizes of methoxyl- or carboxymethyl-PEG5K coated AuNPs. (**a**,**c**) The hydrodynamic diameter, Z_ave_, of 13 nm mPEG-AuNPs and 30 nm cPEG-AuNPs, respectively, determined by dynamic light scattering spectroscopy. (**b**,**d**) TEM imaging of 13 nm mPEG-AuNPs and 30 nm cPEG-AuNPs. (**e**) Sizes and ζ potentials of 13 nm and 30 nm PEG-AuNPs, averaged from five and four batch preparations, respectively. AuNP, gold nanoparticle; PEG, polyethylene glycol; TEM, transmission electron microscopy.

PEGylated AuNPs were produced by linking mPEG-thiol or cPEG-thiol (averaging 5 kDa) to AuNPs with core sizes of 13 and 30 nm, respectively [20,21]. The core sizes, average hydrodynamic sizes, and ζ potentials of 13 and 30 nm mPEG- and cPEG-AuNPs are shown in Figure 2e. Representative DLS results (Figure 2a,c) and TEM images (Figure 2b,d) of 13 nm mPEG-AuNPs and 30 nm cPEG-AuNP analyses are shown. After PEG coating, sizes of AuNPs were significantly increased from uncapped particles. Although there was a greater than 2-fold difference in core size, 30 and 13 nm PEGylated AuNPs differed in hydrodynamic size by only 25%. mPEG-AuNP was about 5% larger than the cPEG-AuNP of the same core size. The degree of PEGylation in prepared PEG-AuNPs can be estimated using an index R, corresponding to the ratio of shell thickness to the diameter of whole PEGylated nanoparticle [7], and the values were found to be averages of 0.62 and 0.32 for 13 nm and 30 nm PEG-AuNPs, respectively, reflecting the higher PEG shell thickness in 13 nm PEG-AuNPs. The mean ζ potential of PEG-AuNPs shows that larger particles were also more negatively charged than small particles of the same coating. cPEG-AuNPs were more negatively charged than mPEG-AuNPs, as expected, but were not substantially different. Results of material characterization were generally in agreement with our previous studies [20,21].

### 2.3. In Vivo Reporter Induction by PEG-AuNPs

**Figure 3:**
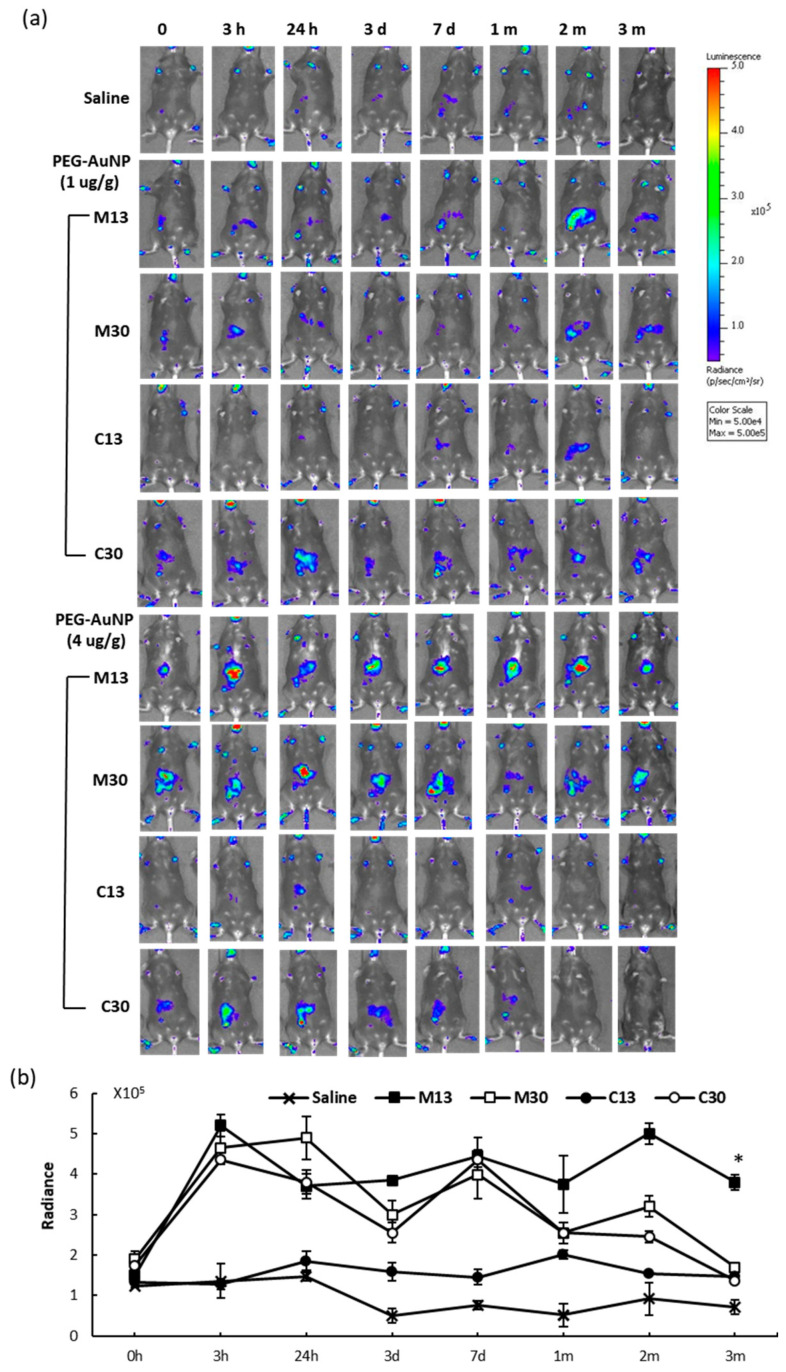
Bioluminescence in reporter mice in response to intravenous PEGylated AuNP injection. (**a**) Low (1 μg/g) or high (4 μg/g) doses of 13 or 30 nm mPEG- (M13, M30) or cPEG-AuNPs (C13, C30) were injected into mice. Images were acquired at the indicated time points for up to 3 months (mos) under the same conditions and imaging settings. (**b**) Mean radiance (photons/sec/cm^2^/sr) of the liver ROI of mice injected with high dose PEG-AuNPs; *n* = 3; * *p* < 0.05 compared to groups at 3 mos post-injection with C13, M30, C30, or saline.

To study NF-κB reporter induction by PEG-AuNPs, with respect to capping and core-particle size, 13 or 30 nm mPEG-AuNPs or cPEG-AuNPs at low (1 μg/g) or high (4 μg/g) doses were injected into Tg mice through tail veins; control mice were injected with saline. The bioluminescent images were acquired at 3 h, 24 h, 3 d, 7 d, 1 mo, 2 mos, and 3 mos using the same conditions and imaging system settings. As in Figure 3a, which shows images of individual mice before and up to 3 mos post-injection, PEG-AuNPs increased bioluminescence in the liver compared to the level before injection and to that in control mice. Variation of endogenous reporter activity in each Tg mouse before nanoparticle injection was noted. Reporter signal was more pronounced at earlier time points, up to 7 days post-injection, than at later times, i.e., 1 to 3 mos post-injection. The results of high dose PEG-AuNPs injection are summarized in Figure 3b. Compared to the radiance on the order of 10^8^ photons/sec/cm^2^/sr induced by TNF-α or LPS, all PEG-AuNPs tested in this study induced bioluminescence at a lower level, in the order of 10^5^ photons/sec/cm^2^/sr in liver. The 13 nm cPEG-AuNPs induced only 1 to 2-fold NF-κB reporter expression at early time points, which is substantially lower compared to the 3 to 7-fold induction by other PEG-AuNPs. After induction by 13 nm mPEG-AuNPs, NF-κB reporter expression remained elevated through the 3 mo time point, while induction by other nanoparticles decreased. There was still an appreciable bioluminescent signal in Tg mice after 3 mos, which indicated continuing PEG-AuNP mediated NF-κB activation in the mouse liver. Thus, we further investigated whether PEG-AuNP-induced NF-κB activation, inflammation, and liver toxicity remained unresolved at the 3 mo time point.

### 2.4. Liver Toxicity and Inflammation in Mice 3 Months after Exposure to PEGylated AuNPs

**Figure 4:**
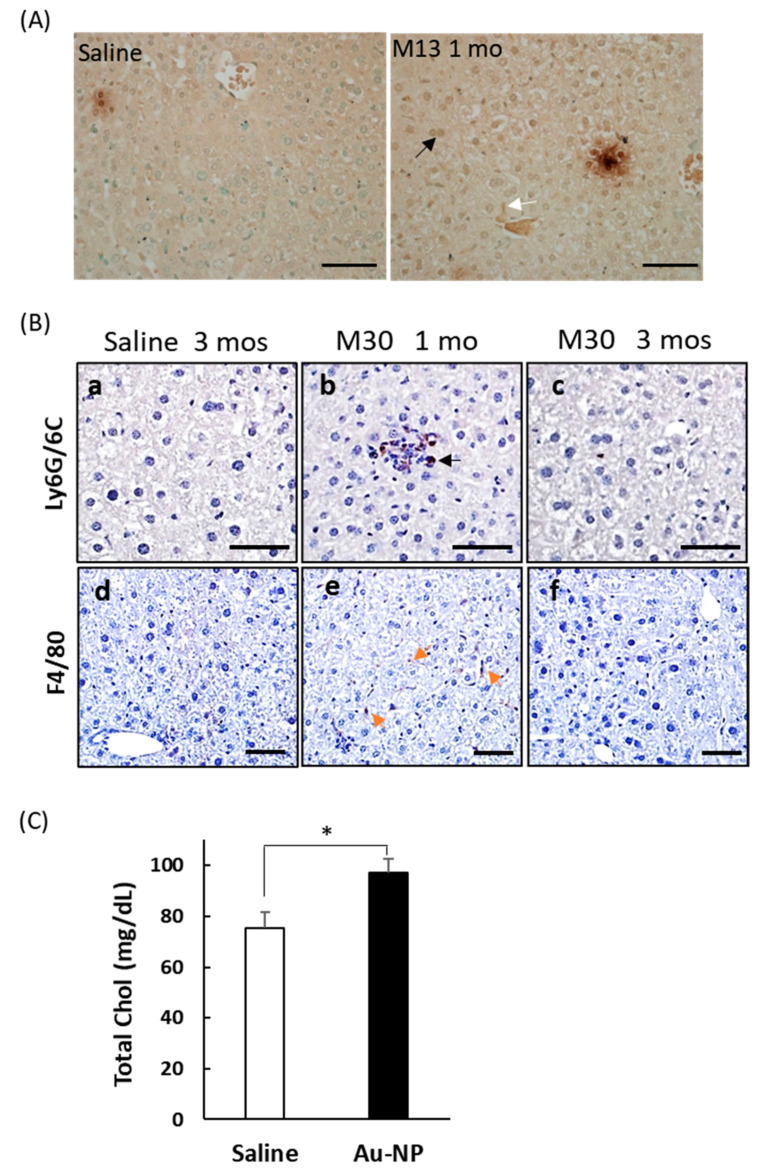
Detection of p65 nuclear localization, Ly6G/6C positive monocytes, and F4/80 positive macrophages in liver, and increased total serum cholesterol. (**A**) p65 immunostaining of liver sections with an anti-p65 antibody and nuclear counterstaining with methyl green. Mouse liver sections from 1 mo post-injection with 13 nm mPEG-AuNPs or with saline are shown. Clusters of dark brown color include positively stained cells and some dye debris. Nuclear-localized p65 was found in Kupffer cells (white arrow) and hepatocytes (black arrow). Scale bar = 50 μm. (**B**) Ly6G/6C positive monocytes (**a**–**c**) and F4/80 positive macrophages (**d**–**f**) were readily detected in liver sections at 1 mo post-injection with 30 nm mPEG-AuNPs (black arrow in **b**; orange arrows in **e**), but rarely detected in liver samples harvested at 3 mos post-injection (**c**, **f**) or in those from saline-injected control mice (**a**,**d**). Scale bar = 50 μm. (**C**) Total serum cholesterol (Total Chol) in Tg mice injected with high doses of cPEG- or mPEG-AuNPs (*n* = 19) was significantly higher than that in the saline-injected group (*n* = 8); * *p* < 0.05.

**Figure 5:**
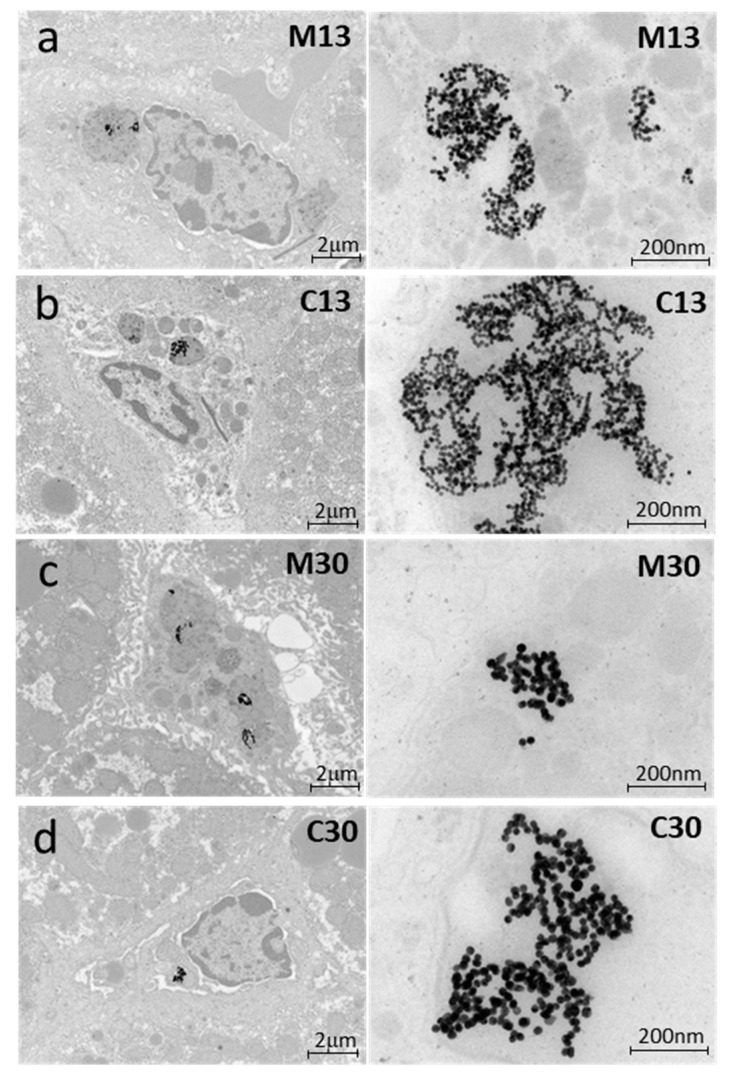
Agglomerates of PEG-AuNPs in liver Kupffer cells at 3 mos post 13 nm mPEG- or cPEG-AuNP injection (**a**,**b**), or by 30 nm mPEG- or cPEG-AuNP injection (**c**,**d**). Higher magnification images are shown at right.

To confirm that NF-κB was activated in the mouse liver, as indicated by reporter imaging, immunohistochemical staining of liver sections with an anti-p65 antibody was performed, as p65/RelA translocation to the cell nucleus is a well-established indicator of NF-κB activation. The results showed increased nuclear p65 staining in mouse liver sections 1 mo after 13 nm mPEG-AuNP injection (Figure 4A). To confirm inflammation in liver sections, we performed immunostaining for Ly6G/6C to identify the presence of monocytes and F4/80 to identify activated Kupffer cells. Both monocytes and F4/80-positive Kupffer cells were most frequently found in mouse liver tissue 1 mo after mPEG-AuNP injection, and were rarely detected in liver sections from saline-injected controls or 3 mos after mPEG-AuNP injection (Figure 4B), confirming that elevated liver inflammation induced by PEG-AuNPs gradually resolved, in accordance with imaging results. After 3 mos, mice were sacrificed, blood serum was collected, and levels of liver enzymes were measured. We observed an increase in alanine aminotransferase (ALT) level following PEG-AuNP injection, although the difference was not statistically significant (*p* = 0.11) (data not shown). All PEG-AuNP injected mouse data in combination indicated increased total cholesterol compared to saline controls (Figure 4C), yet the level remained within the normal range of C57BL/6 mice. This result suggests that sustained inflammation caused by PEG-AuNP contributed to changes leading to hepatic dysfunctions. We collected left front lobe liver tissue and processed it for TEM to determine whether PEG-AuNPs remained in liver 3 mos after injection. As shown in Figure 5, agglomerates of 13 nm or 30 nm mPEG- or cPEG-AuNPs were found primarily enclosed in intracellular lysosomes of Kupffer cells [22]. There were substantially higher numbers of 13 nm AuNPs than 30 nm particles because an equal weight of each particle type per mouse body weight was administered. The appearance of particles as agglomerates in larger lysosomes after 3 mos may be due to many episodes of sorting and fusion of small lysosomes in macrophages and Kupffer cells.

### 2.5. Enhanced Collagen Deposition in Liver Tissues after PEG-AuNP Injection

**Figure 6:**
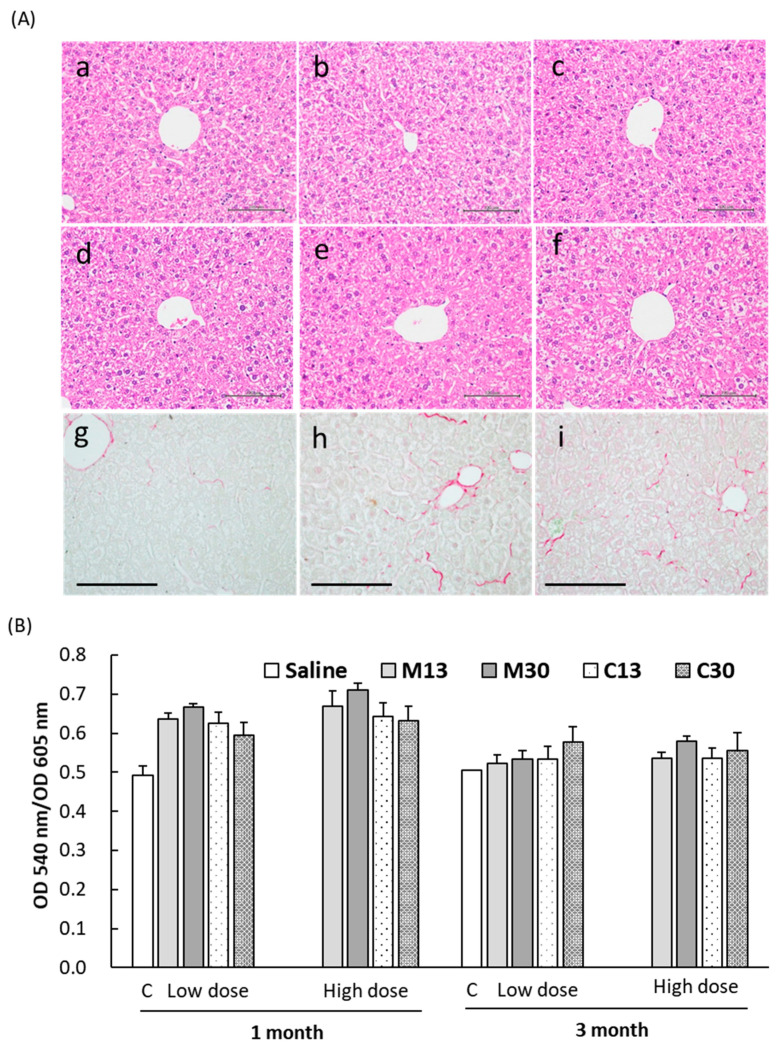
Pathology and collagen deposition in liver tissue from a PEG-AuNP-injected mouse. (**A**) H&E (**a**–**f**) and Sirius Red (**g**–**i**) staining of liver sections of control (**a**,**d**,**g**), and at 1 mo (**b**,**e**,**h**), and 3 mos (**c**,**f**,**i**) after injection with 30 nm mPEG-AuNPs (4 μg/g). There were no significant abnormalities due to nanoparticle injection in liver sections (**a**–**f**). Siris Red staining of sinusoidal microvasculature increased at 1 mo and 3 mos after PEG-AuNP injection compared to saline-injected controls (**g**–**i**). Scale bar = 100 μm. (**B**) Semiquantitative estimation of collagen content in liver sections via the ratio of dye absorbances of Sirius Red (540 nm) and Fast Green (605 nm) after dye extraction from stained sections. C: saline injection.

We also investigated whether pathological abnormalities were caused by PEG-AuNP injection. H&E staining showed that PEG-AuNP injection did not result in significant abnormalities in the mouse liver at 1 mo or 3 mos post-injection (Figure 6A). Some minor liver pathologies relating to PEG-AuNP injection included clusters of infiltrating cells that were sporadically found in the liver 1 mo after injection, and suspicious necrotic hepatic cells and mildly increased glycogen levels at 3 mos; however, none of these changes were significant. Chronic liver inflammation is an established cause of liver steatosis, fibrosis, and potentially cirrhosis, therefore, we stained liver sections to assess the presence of collagen. As shown in Figure 6A (g–i), PEG-AuNP injection caused a significant increase in collagen staining at 1 mo and 3 mos compared to saline-injected controls. Sinusoidal capillaries, in addition to larger blood vessels, showed strong collagen staining at 1 mo that was also confirmed via Masson trichrome staining (not shown). At 3 mos, the staining of collagen by Sirius Red at sinusoidal capillaries was less intense than at 1 mo, but collagen staining in hepatocytes was increased at 3 mos compared to that of 1 mo or controls. Quantification of Sirius Red staining results using dye extraction from the specimen revealed higher collagen content at 1 mo than at 3 mos, reflecting a reduction of collagen deposition in sinusoidal vessels at 3 mos (Figure 6B).

## 3. Discussion

### 3.1. PEGylation Reduces Acute Toxicity of AuNPs

Applications using naked AuNPs as drug or gene carriers were limited, in part, due to associated hepatotoxicity and rapid elimination by the RES [23]. PEG coating of AuNPs allows adsorption of serum protein and significantly improves the particle distribution in blood while reducing inflammation and liver toxicity [1]. Thus, only AuNPs coated with PEG or another polymer will be considered for clinical applications. Previously, only the short-term toxicity of PEG-AuNPs was studied [1,11], and primarily in comparison with naked AuNPs. Direct comparison of different PEG coatings to AuNPs in their ability to mitigate long-term toxicity has not been reported. In this study we examined liver toxicity after intravenous injection of PEGylated AuNPs, with data collected for up to 3 mos and monitored NF-κB activation using reporter Tg mice. According to ICH guideline M3(R2), data from rodents tested over 3-mo period are suitable to support subsequent clinical assessments, in phase II or III trials, of the toxicity of small molecules intended for clinical use within a 3-mo time frame [24,25]. Two PEG-AuNP doses, 4 and 1 μg per gram of body weight, were used in this study because they were comparable to doses used previously [23] and higher than those of other studies showing liver toxicity [2,11]. Nevertheless, imaging, pathology, and blood chemistry results were in agreement, demonstrating that PEG-AuNP injection did not lead to significant liver toxicity after 3 mos. The data at 3 mos that showed an absence of liver pathology is particularly important for drug development because 1-mo data showed liver inflammation induced by PEG-AuNPs. Future 6-mo to 1-year data from rodents will further facilitate drug development from the perspective of regulatory toxicology [25]. Furthermore, cPEG-AuNP injection resulted in even less reaction in the liver compared to that associated with mPEG-AuNPs; this is a promising result that is worthy of further study.

### 3.2. Reporter Mice for Monitoring Liver Inflammation

NF-κB luciferase Tg mice have become a useful and commercially available molecular imaging tool for tracking in vivo NF-κB activation in cancer, genetic issues, infection, immunity, and toxicology. The dual reporter mouse described here allowed the nuclear imaging capability and afforded additional flexibility in situations where precise reporter quantification in 3D or via dual imaging modality is needed. One of the advantages of longitudinal imaging is that the baseline activity of each mouse and its response after the challenge is revealed, as opposed to pooling results from a specific time point and requiring the use of many more animals. Although some Tg mice displayed higher baseline NF-κB activity than others, PEG-AuNP injection always increased the bioluminescent output well above baseline in each animal (Figure 3). We also observed that the bioluminescence acquired in saline-injected control mice was gradually reduced during the study period, with mice assessed starting at 8 weeks of age until 22 weeks of age. Reduction of reporter activity in older mice is consistent with reports on the age-dependence of endogenous NF-κB activity.

AuNPs of 10–50 nm in diameter were shown to have the longest retention in circulation [26,27]. We did not observe significantly different outcomes with 13 versus 30 nm PEG-AuNPs, likely due to increased and similar final particle sizes, via PEG coating, of 40–43 nm and 47–50 nm, respectively. The distinct differences between “synthetic identity” and “biological identity” of nanoparticles in blood are well-recognized [8]. The mechanism by which changes in surface charge result in altered biological identity in vivo remain unclear; these alterations may include changes in specificity of binding to serum proteins, altered uptake by the MPS and RES, and modifications in the triggering of inflammatory responses by cells. Nanoparticle coating with cPEG rather than mPEG would render a more negative charge surface and, in theory, would increase serum protein adsorption and subsequent uptake by the RES [4]. NF-κB induction by the PEG-AuNPs tested in this study was relatively mild compared to induction by LPS or TNF-α; still, we observed distinctions in reporter response induced by mPEG versus cPEG coatings. The reporter expression induced by 13 nm mPEG-AuNPs remained significantly elevated at 3 mos, in contrast to the reduced reporter expression in controls and in other particle-injected mice at the same time point. Interestingly, the 13 nm cPEG-AuNPs led to the least NF-κB induction at early time points, when at least 3-fold induction was induced by all other types of PEG-AuNPs included in the study. The differences in reporter expression by particle size and type of PEG coating were not reflected by other assays, such as liver enzymes or pathology ones, likely due to the higher sensitivity of the NF-κB bioluminescence reporter assay compared to other methods in detecting inflammation and/or due to the compounded NF-κB activation response in liver as a result of continuing Kupffer cell activation.

### 3.3. Chronic Low-Grade Hepatic Inflammation Induced by PEG-AuNPs

The exposure and default accumulation of nanoparticles in the RES and MPS of the liver may expose hepatic tissues to delayed toxicity or result in chronic inflammation. Hepatocyte death and impairment in liver sinusoidal endothelial cells (LSEC), a major component of the RES, are often accompanied by features that herald the transformation from clinically benign obesity-related fatty liver, or steatosis, to the more serious non-alcoholic steatohepatitis (NASH) [28,29,30,31]. Our results showed that PEG-AuNP led to hepatic inflammation at an earlier time point with an increase in monocytes and F4/80-positive Kupffer cells, which were rarely found in liver sections at 3 mos (Figure 4B), indicating that the inflammation induced by PEG-AuNPs gradually resolved after 1 mo. By using Masson trichrome (not shown) and Sirius Red staining, collagen deposition was highest at 1 mo after nanoparticle injection, predominantly in liver sinusoidal endothelial cells. The collagen staining of sinusoids decreased at 3 mos compared to staining at 1 mo, suggesting that early increases in collagen at sinusoids were due to inflammation triggered by PEG-AuNPs. In contrast, the increased intensity of collagen dye in hepatocytes at 3 mos compared to 1 mo was likely due to a phenotype change in hepatocytes resulting in different protein/lipid composition that affected dye adsorption.

We observed an increase in total cholesterol in mice 3 mos after PEG-AuNP injection without induction of the liver enzymes aspartate aminotransferase (AST) and ALT. Liver enzyme activity, in particular of AST, was previously shown to not be specific to nanoparticle-induced injury [11]. Sustained liver inflammation and oxidative stress can lead to cholesterol dysregulation and hypercholesterolemia; therefore, more research is needed to address whether increased cholesterol caused by PEG-AuNPs in liver increases risk of liver steatosis. Several studies have addressed the involvement of various metal nanoparticles in liver steatosis [9]. For example, naked AuNPs’ (15 nm) injection aggravated hepatic damage in a mouse steatohepatitis model by stimulating the inflammatory response and accelerating stress-induced apoptosis. Less injury resulted from injecting mPEG-AuNPs than from naked AuNPs, and the injury was also less in mice that did not have concurrent liver stress [32]. Silver (Ag)NPs led to pro-inflammatory activation of Kupffer cells in the liver, enhancement of existing hepatic inflammation, and promotion of fatty liver disease [33]. Although PEG and other polymer conjugated Au- and Ag-NPs have been studied as potential carriers of nanodrugs for treatment of various liver diseases [34,35,36], exposure to Au- or AgNPs likely aggravates nonalcoholic fatty liver disease (NALFD) [32]. Our results showing increased cholesterol levels following PEG-AuNP exposure is an alarming issue regarding the long-term safety of treatment via PEG-AuNPs.

### 3.4. Study Limitations

The dual reporter Tg mice described here were sufficiently responsive to NF-κB induction by LPS or TNF-α, producing 20 to 60-fold induction with a strong light output of 10^8^–10^9^ photons/s/cm^2^/sr (Figure 1), and by PEGylated AuNPs with up to 7-fold induction (Figure 3); however, the photon output induced by PEG-AuNPs was at least 1000-fold less than that induced by LPS or TNF-α. In the context of low induction, endogenous basal NF-κB activity of each mouse affects pooled imaging results. Our data demonstrated that longitudinal imaging of individual mice allowed clearer assessment of PEG-AuNP induction of NF-κB activity. Both innate and adaptive immunity are known to decline with age [31,37], and we observed a gradual reduction in NF-κB activity in saline-injected controls over time, which may be related to a diminished NF-κB response in older animals. Thus, long-term observation using reporter animals would facilitate the study of varied endogenous activity and age-related decline in NF-κB response. On the other hand, hepatic tissues and the MPS and RES are also subject to alteration throughout long study periods.

As a study of the potential toxicity of nanoparticles, we used 4 and 1 μg/g PEG-AuNP doses, which are higher than those likely to be used in real-world clinical applications. Lower doses of PEG-AuNPs would likely elicit less inflammation and be better tolerated in vivo than the those assessed in this study. Our results demonstrated a lack of significant pathology and very low NF-κB induction at 3 mo following nanoparticle injection, thereby indicating that PEG-AuNPs lead to minimal long-term hepatotoxicity. However, increased total cholesterol in mice, although still within the normal range, cannot be ignored, as it indicates that PEG-AuNP exposure impacts liver function. Additional longer-term studies will be needed to determine the consequences of mild NF-κB induction by AuNPs trapped in the MPS and RES.

## 4. Materials and Methods

### 4.1. Chemicals and Reagents

All chemicals not specified elsewhere were purchased from Sigma-Aldrich (St. Louis, MO, USA), TNF-α (Peprotech, Cranbury, NJ, USA), anti-Ly-6G/6C antibody (BD Biosciences, La Jolla, CA), lipopolysaccharide (LPS) (Sigma-Aldrich), D-luciferin (Perkin Elmer, Waltham, MA, USA), anti-p65 antibody (Abcam, Cambridge, MA, USA) were used.

### 4.2. PEG-AuNP Preparation and Characterization

AuNPs with a core diameter of 13 nm or 30 nm were produced using the Turkevich method, which involves reduction of Au from auric acid (HAuCl_4_) by sodium citrate with slight modifications [20,38]. For the synthesis of 13 nm AuNPs, auric acid aqueous solution (1.0 mM, 500 mL) was heated to boil with a vigorous stir. Sodium citrate solution (38.8 mM, 62.5 mL) was subsequently added for reducing Au(III) to Au^0^. This solution was boiled with stirring for another 10 min until its color turned into burgundy. For the synthesis of 30 nm AuNPs, auric acid aqueous solution (5.0 mM, 25 mL) was mixed with NaOH solution (1.0 M, 0.3mL) and adjusted to 500 mL with double deionized water. The mixture was heated to boil with a vigorous stir and sodium citrate solution (5%, 5.2 mL) was subsequently added for reducing reaction. After the reaction, the solution was cooled to room temperature in 2 h. The hydrodynamic size and ζ potential of PEG-AuNPs were determined by dynamic laser light scattering (DLS) measurements (Zetasizer Nano ZS, Malvern Instruments, Worcestershire, UK) using a He-Ne laser (633 nm) at an angle of 173°. Methoxyl-poly(ethylene glycol)-thiol (mPEG; NOF Co., Tokyo, Japan) and carboxymethyl-poly(ethylene glycol)-thiol (cPEG; Laysan Bio, AL, USA) with molecular weights averaging 5 kDa were conjugated to the surfaces of AuNPs through a ligand exchange procedure, as previously described [20,21]. The PEGylated AuNPs were then resuspended in ddH_2_O and assessed for endotoxin contamination to ensure the particle preparations did not contain detectable endotoxin (i.e., less than 0.48 EU/mL).

### 4.3. Generation of the NF-κB Reporter Tg Mouse

An NF-κB reporter vector was designed to express firefly luciferase (fLuc) and a truncated herpes simplex virus type 1 thymidine kinase (HSV1Δtk) fusion protein. Ten copies of an NF-κB responsive element (RE) was constructed by linking five AAGGGACTTTCCGGGAATTT repeats with the underlined NF-κB consensus sequence. A TATA box and Cytomegalovirus (CMV) minimal enhancer were included prior to the coding sequences for fLuc and the HSV1Δtk fusion protein. The function of individual reporters was assessed and validated via imaging in our previous paper describing a tri-fusion reporter [19].

All animal experiments were conducted in accordance with accepted standards of animal care and approved by the Institutional Animal Care and Use Committee (NHRI-IACUC-103107A, 15 August 2014) of the National Health Research Institutes (NHRI), Taiwan. The NF-κB reporter Tg mouse model was generated on a C57BL/6 strain background at the NHRI transgenic core facility using a 3.1 kb ApaL1/MluI-digested fragment as the NF-κB vector. Six transgenic founders were analyzed, and three lines were maintained (#27, #17, and #12). Genotyping of transgene-positive mice was performed by PCR using the primers GGACCTATGATTATGTCCGG and GCCCGGTTTCAATTTGGACTTTC. This study used only mice from line #17.

### 4.4. PEG-AuNP Injection and In Vivo Optical Imaging

Eight-week-old B6 NF-κB reporter Tg mice were randomized into groups (5–7 mice per group) and received either a high (4 μg per gram of body weight) or low (1 μg/g) dose of mPEG- or cPEG-AuNPs through tail vein injection. Before injection, normal saline was used to dilute the PEG-AuNPs to equalize injection volume to 100 μL per mouse. Bioluminescence imaging was performed using an IVIS Spectrum series animal imager (Caliper Life Sciences, Hopkinton, MA, USA). Mice were anesthetized with 2.5% isoflurane followed by intraperitoneal injection of D-luciferin (150 mg/kg). Images were acquired 10 min after injection of the substrate and were repeated until the signal was attenuated or after 30 min.

### 4.5. Transmission Electron Microscopy

Prepared PEG-AuNPs were drained and dried on copper grids for TEM measurement (H7650 Hitachi, Tokyo, Japan). Both AuNPs and PEG-AuNPs were counted using SigmaScan Pro 5.0 (Systat software, San Jose, CA, USA) and data from at least 300 nanoparticles was averaged for determining the particle sizes. The left lobe of mouse liver was harvested and fixed overnight in 2% paraformaldehyde and 2% glutaraldehyde. After the liver tissue was washed with PBS, samples were postfixed for 1 h in 2% OsO_4_, then washed in ddH_2_O, and finally dehydrated stepwise in increasing concentrations of ethanol. Resin embedding, sectioning to 100 nm slices by ultramicrotome, and TEM imaging and measurements were contracted to Bio MA-Tek Taiwan, which used a Hitachi HT7700 TEM system.

### 4.6. Histological and Blood Chemistry Analyses

Mouse livers were fixed in 10% formalin overnight. Paraffin-embedded sections were stained with hematoxylin and eosin (H&E) or assessed for collagen content by following the manufacturer’s protocol [39] in use of a Sirius Red/Fast Green collagen staining kit (#9046, Chondrex, Redmond, WA, USA). In short, deparaffinized tissue sections were incubated with the reaction buffer containing both Sirius Red and Fast Green dyes for 30 min. After incubation, the sections were rinsed repeatedly with water until the water ran clear. The Sirius Red dye binds to collagen and the Fast Green dye binds to non-collagenous proteins. After the bright field images were captured, both dyes were eluted from the sections using extraction buffer and the absorption of eluted dye at 540 nm (Sirius Red) and 605 nm (Fast Green) was measured. The ratio of optical density (OD) values was used as a semiquantitative estimation of collagen and non-collagenous protein in each section. Blood chemistry analysis was performed using the DRI-CHEM 3500s (Fuji Film, Tokyo, Japan) and the Fuji Dry-Chem Slide (TCHOP-P III).

### 4.7. Statistical Analysis

The data are presented as mean ±SEM with differences between groups analyzed by Student’s two tailed *t*-test. *p* < 0.05 was considered as statistically significant.

## 5. Conclusions

In this study, we addressed the long-term hepatotoxic effects of intravenous mPEG- or cPEG-AuNP injection by monitoring the inflammatory response of the NF-κB reporter mouse over 3 mos. Transient NF-κB reporter induction and liver inflammation were readily detected by animal imaging methods, occurred at early time points, and were reduced by 3 mos after injection, as indicated by the lack of liver abnormalities based on conventional pathology assessment; however, higher serum cholesterol and increased collagen staining in liver tissue sections were observed. As PEG-AuNPs and most metal-based nanoparticles accumulate in the liver, further study is needed to assess the potential for increased long-term risk of developing steatohepatitis following nanoparticle exposure.

## Abbreviations

PEG	Polyethylene Glycol
DLS	Dynamic Laser Light Scattering
AuNP	Gold Nanoparticles
HSV1tk	Herpes Simplex Virus type 1 Thymidine Kinase
PET	Positron Emission Tomography
SPECT	Single Photon Emission Computed Tomography
MPS	Mononuclear Phagocyte System
RES	Reticuloendothelial System
mPEG	Methoxyl-poly(ethylene glycol)-thiol
cPEG	Carboxymethyl-poly(ethylene glycol)-thiol
TEM	Transmission Electron Microscope
ICH	International Council for Harmonisation of Technical Requirements for Pharmaceuticals for Human Use
